# Gender Differences in Infant Mortality and Neonatal Morbidity in Mixed-Gender Twins

**DOI:** 10.1038/s41598-017-08951-6

**Published:** 2017-08-18

**Authors:** Dongying Zhao, Lile Zou, Xiaoping Lei, Yongjun Zhang

**Affiliations:** 1 Xinhua Hospital, Shanghai Jiao Tong University School of Medicine, Shanghai, China; 2 Department of Histology and Embryology, Southwest Medical University, Luzhou, Sichuan China; 3 Department of Neonatology, Affiliated Hospital of Southwest Medical University, Luzhou, Sichuan China

## Abstract

In the present study, we aimed to explore gender differences in infant mortality and neonatal morbidity in mixed-gender twin pairs. Data were obtained from the US National Center for Health Statistics Linked Birth-Infant Death Cohort. A total of 108,038 pairs of mixed-gender twins were included in this analysis. Among the mixed-gender twins, no significant difference in the odds of fetal mortality between male twins (1.05%) and female co-twins (1.04%). However, male twins were at increased odds of neonatal mortality (adjusted OR 1.59; 95% CI 1.37, 1.85) and overall infant mortality (adjusted OR 1.43; 95% CI 1.27, 1.61) relative to their female co-twins. Congenital abnormalities (adjusted OR 1.38; 95% CI 1.27, 1.50) were identified significantly more frequently in male than female twins. Moreover, increased odds of having low 5-minute Apgar score (<7) (adjusted OR 1.15; 95% CI 1.05, 1.26), assistant ventilation >30 minutes (adjusted OR 1.31; 95% CI 1.17, 1.47), and respiratory distress syndrome (adjusted OR 1.45; 95% CI 1.26, 1.66) were identified in male twins relative to their female counterparts. The results of our study indicated that in mixed-gender twin pairs, the odds of infant mortality and neonatal morbidity were higher in male twins than their female co-twins.

## Introduction

Over the past several decades, male subjects have been found to be at increased risk of mortality and morbidity relative to their female counterparts in not only the perinatal period but also throughout the entire lifespan^1–4^. Moreover, male gender has been found to be associated with increased risk of prematurity, respiratory distress syndrome (RDS) and intrauterine growth restriction^3–6^. However, some previous studies have reported the identification of lower rates of mortality in male than female infants due to disparities in the treatment of girls in some Asian countries^7, 8^. Most of these studies were conducted in singleton populations, the results of which may have been influenced by different intrauterine, household and social factors. In contrast, the genetic background, intrauterine and household environments of mixed-gender twin pairs are more similar than those of singleton pairs from different families. Comparing male twins and their female co-twins in mix-gender twin pairs can offer better opportunities to expose sex-related differences in infant mortality and neonatal morbidity.

Several studies have been conducted to explore the relationship between sex and prenatal outcomes in twin populations^3–5^. Most of these studies have compared male-male or female-female twin pairs^3, 5^. One study compared male twins and female co-twins, but only focused on the neonatal mortality and morbidity^4^. Research on the inter-sex differences in infant mortality and neonatal morbidity in mixed-gender twins remains limited. Thus, in the present study, we investigated the effect of sex on infant mortality and neonatal morbidity in a population of mixed-gender twins.

## Methods

This study was based on data from the US National Center for Heath Statistic’s (NCHS) 1995–2004 matched multiple birth dataset^9^. The NCHS-matched multiple birth data contain information on maternal and pregnancy characteristics and perinatal and infant mortality for all multiple births that occurred from 1995–2004 in the United States. The twin data were abstracted from live birth and infant death certificates. The two babies composing twin pair were linked based on a 3-stage matching algorithm developed by the NCHS. The details of this algorithm have been described elsewhere^10^. This algorithm has been validated and found to be 99% accurate in linking 2 infants to a twin births^10^. Data on maternal demographic characteristics, obstetric history, current pregnancy, labor and delivery complications, and birth outcomes are available in these files. Since these data are publicly available in anonymized form, the study was exempted from ethical approval by the Institutional Review Board of Xinhua Hospital, Shanghai Jiao Tong University School of Medicine.

The following obstetric and neonatal data were collected from the live birth certificates: maternal race, age, smoking status during pregnancy, education, marital status, pregnancy complications, labor complications and caesarean section status and the fetal sex, mode of delivery, gestational age, birth weight (grams), and morbidities, congenital anomalies, RDS, need for assistant ventilation >30 minutes and low 5-minute Apgar score (<7). In addition, fetal deaths occurring after 20 completed gestational weeks were registered as stillbirths, and neonatal and infant mortalities were defined as deaths occurring during the first 27 days and first 364 days of life, respectively.

**Figure 1:**
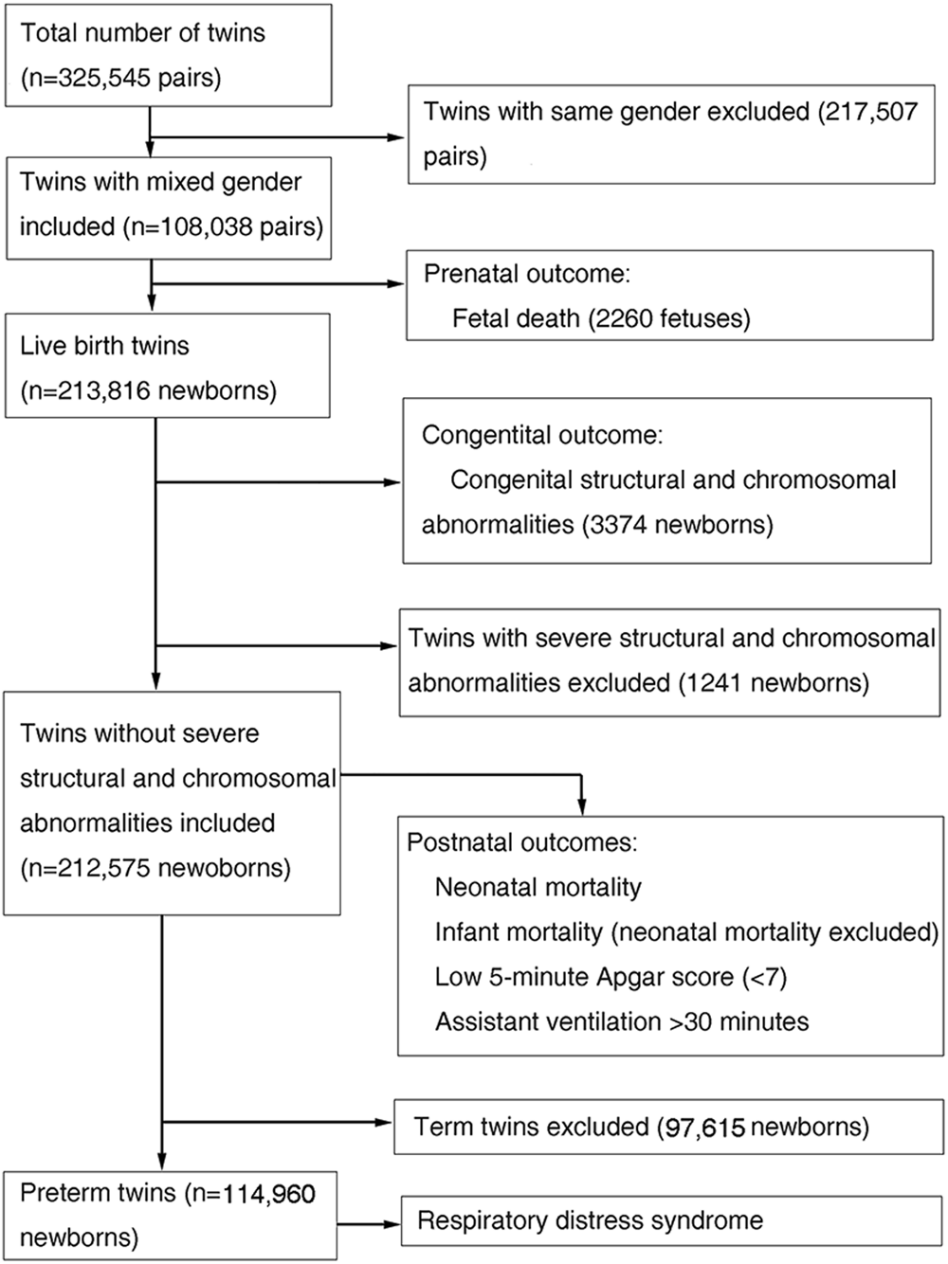
Flow diagram. The U.S. National Center for Health Statistics Linked Birth-Infant Death Cohort, Detail Matched Multiple Birth datasets from 1995–2004.

In total 325,545 pairs of twins were included in the NCHS-matched multiple birth data. After excluding 217,505 pairs of same-gender twins, we examined gender differences in fetal mortality in 108,038 pairs of mixed-gender twins. Within the 213,816 live-born twins, the rates of congenital structural and chromosomal abnormalities were compared between male twins and female co-twins. Babies with severe structural and chromosomal abnormalities may have elevated risk of mortality and morbidity after birth; thus, for this analysis, we excluded 1241 newborns with encephalitis, spina bifida, meningocele, microcephalus, severe heart malformations and chromosomal abnormalities to further explore gender differences in infant mortality and neonatal morbidity. The definition of RDS included clinical evidence of respiratory difficulties (tachypnea, retraction, grunting and cyanosis) and radiographic appearance of RDS (low volume lungs with a diffuse reticulogranular pattern and air bronchograms)^11^. Because RDS was most frequently identified in preterm infants, we excluded term twins to evaluate the association between sex and RDS. (Fig. 1) Moreover, the results of a previous study indicated that the effect of race on prenatal health was predominantly moderated by social mechanisms^12^. To avoid the influence of race, we performed a stratified analysis to assess the effect of this variable.

### Statistical analysis

The design of the present analysis was an inter-matched case-control study, the two inter-matched babies came from the same pregnancy. Most of the confounders were matched, except for birth weight, birth order and methods of delivery (the first baby was vaginal delivery, but the second one was cesarean section in partial twins). All outcomes were coded as dichotomous variables. Cochran-Mantel-Haenszel Chi-square was used to assess the differences of the unmatched confounders. An univariate conditional logistic regression analysis was performed to calculate the crude odds ratios (ORs) and 95% confidence intervals (CIs) of the differences in outcomes between the male and female members of mix-gender twins. A multiple conditional logistic model was constructed to adjust for unmatched confounders. A p-value < 0.05 was considered statistically significant. All analyses were carried out using SAS version 9.2 (SAS Institute Inc, Cary, NC).

## Results

### Demographic and obstetrical characteristics

Table 1 presents the baseline characteristics of the mothers of mixed-gender twins. Most of the twin’s mothers were white (78.0%), and the mean maternal age was 29.5 years. Approximately 84.4% mothers had at least a high school level of education. Maternal smoking was identified in 9.1% of the women. The majority of women underwent caesarean section (63.2%), while the rates of pregnancy and labor complications were 18.5% and 13.8%, respectively. Table 2 shows that the male twins have bigger birth size (2200 g ± 796 g vs 2094 g ± 778 g, P < 0.001) and more chance to delivery firstly than their female co-twins.

**Table 1:** Baseline Characteristics of the Mothers of Mixed-Gender Twins.

Maternal characteristics	
Maternal Race (n, %)	
White	84,215 (78.0)
Black	20,382 (18.9)
Others	3441 (3.2)
Maternal age (Mean, SD)	29.5 (6.0)
Maternal smoking status during pregnancy (n, %)	
No smoking	78979 (73.1)
Smoking	9867 (9.1)
Unknown	19192 (17.8)
Maternal education (n, %)	
<12y	15,162 (14.0)
12–15y	56,540 (52.3)
>15y	34,583 (32.0)
Unknown	1753 (1.6)
Marital status (n, %)	
Married	78,599 (72.8)
Unmarried	29073 (26.9)
Unknown	366 (0.3)
Weight gain during pregnancy (Kg)	16.6 (7.2)
Parity	
0	42,798 (39.6)
≥1	65,240 (60.4)
Pregnant complications^1^ (n, %)	19,949 (18.5)
Labor complications^2^(n, %)	14,892 (13.8)
Methods of delivery (n, %)	
Cesarean section	68,256 (63.2)
Vaginal	27989 (25.9)
Vaginal to Cesarean section^3^	4714 (4.4)
Unknown	7079 (6.6)

^1^Defined as one or more of the following diseases or conditions: anemia (HCT <30 or Hb <100 g/L), cardiac disease; acute or chronic lung disease, diabetes, hydramnios or oligohydramnios, hemoglobinophathy, chronic or pregnancy-associated hypertension, and renal disease. ^2^Defined as one or more of the following diseases or conditions: premature rupture or membrane >12 hours, abruption placenta, placenta previa, precipitous or prolong labor, dysfunctional labor, seizure during labor, cephalopelvic disproportion, and excessive bleeding. ^3^The first babies were vaginal delivery and the second ones by cesarean section.

**Table 2:** The Unmatched Factors between the Male and the Female Co-twins in Mixed Gender Twins.

	The male twins	The female co-twins	***P***
Birth weight (g, Mean, SD)	2200 (796)	2094 (778)	<0.001
Birth order			0.02
First	48,650 (45.0)	48,013 (44.5)	
Second	48,013 (44.5)	48,650 (45.0)	
Unknown	11,375 (10.5)	11,375 (10.5)	
Methods of delivery			0.88
C-section	71,046 (65.8)	70,883 (65.6)	
Vaginal	29,913 (27.7)	30,076 (27.8)	
Unknown	7079 (6.6)	7079 (6.6)	

### Perinatal outcomes

Among the 108,038 pairs of mixed-gender twins included in this study, the rate of fetal mortality was 1.05% (2260/216,076), and no difference was identified between male twins (1.05%) and female co-twins (1.04%) (OR 1.01, 95% CI 0.91, 1.13). Among live-born twins, 3374 (1.58%) newborns had a congenital structure or chromosomal abnormality. The rate of congenital abnormalities (adjusted OR 1.38; 95% CI 1.27, 1.50) was higher in males than their female co-twins. The overall rate of infant mortality was 2.42% among mixed-gender twins without severe structural or chromosomal abnormalities. The odds of mortality were ignorantly greater among male twins than their female co-twins (adjusted OR 1.43; 95% CI 1.27, 1.61). Similar trends were identified for neonatal mortality (adjusted OR 1.59; 95% CI 1.37, 1.85) and infant mortality after 28 days (adjusted OR 1.18; 95% CI 1.00, 1.40). Moreover, the odds of low 5-minute Apgar score (<7) (adjusted OR 1.15; 95% CI 1.05, 1.26) and assistant ventilation >30 minutes (adjusted OR 1.31; 95% CI 1.17, 1.47) were significantly greater in males than females. After excluding term twins, 6019 (5.17%) of the total 116,531 premature newborns developed RDS, and male twins were also at greater odds of RDS than their female co-twins (adjusted OR 1.45; 95% CI 1.26, 1.66) (Table 3).

**Table 3:** Mortality and Morbidity in Mixed-Gender Twins: Comparison Between Male and Female Co-twins.

Outcomes	The male twins (n, %)	The female twins (n, %)	OR (95%CI)	aOR (95%CI)
Fetal mortality	1134 (1.05)	1126 (1.04)	1.01 (0.91, 1.13)	NA
Congenital abnormality	1867 (1.75)	1507 (1.41)	1.38 (1.27, 1.50)	NA
Infant mortality	2675 (2.52)	2412 (2.27)	1.30 (1.19, 1.42)	1.43 (1.27, 1.61)
*Neonatal mortality*	*2152* (*2.03*)	*1964* (*1.85*)	*1.35(1.21*, *1.51*)	*1.59* (*1.37*, *1.85*)
*Infant mortality after 28 days*	*523* (*0.49*)	*448* (*0.42*)	*1.17* (*1.03*, *1.34*)	*1.18* (*1.00*, *1.40*)
Low 5-minute Apgar score (<7)	3612 (3.40)	3428 (3.22)	1.09 (1.00, 1.19)	1.15 (1.05, 1.26)
Assistant ventilation >30 minutes	4318 (4.06)	3897 (3.67)	1.42 (1.31, 1.54)	1.31 (1.17, 1.47)
Respiratory distress syndrome	3046 (5.30)	2721 (4.73)	1.52 (1.33, 1.72)	1.45 (1.26, 1.66)

OR: Odds Ratio; aOR: Odds Ratio adjusted by birth weight, birth order and methods of delivery.

### Subgroup Analyses

We further stratified the study subjects by race to explore the sex differences in infant mortality and perinatal morbidity within each race group. Similar results were identified in the white and black populations (Table 4).

**Table 4:** Race-stratified Analysis of Mortality and Morbidity in Male and Female Co-twins in Mixed-Gender Twin Pairs.

Race	Outcomes	The male twins (n, %)	The female twins (n, %)	OR (95%CI)	aOR (95%CI)
White	Fetal mortality	725 (0.86)	716 (0.85)	1.02 (0.89, 1.17)	NA
	Congenital abnormality	1460 (1.75)	1180 (1.41)	1.36 (1.24, 1.49)	NA
	Infant mortality	1638 (1.97)	1457 (1.76)	1.35 (1.21, 1.50)	1.51 (1.34, 1.70)
	*Neonatal mortality*	*1351* (*1.63*)	*1217* (*1.47*)	*1.39* (*1.21*, *1.59*)	*1.58* (*1.36*, *1.83*)
	Low 5-minute Apgar score (<7)	2336 (2.82)	2175 (2.62)	1.14 (1.05, 1.23)	1.17 (1.08, 1.28)
	Assistant ventilation >30 minutes	3390 (4.09)	2997 (3.61)	1.51 (1.38, 1.65)	1.42 (1.28, 1.57)
	Respiratory distress syndrome	2464 (5.6)	2158 (4.90)	1.63 (1.45, 1.82)	1.51 (1.34, 1.71)
Black	Fetal mortality	374 (1.83)	376 (1.84)	0.99 (0.80, 1.22)	NA
	Congenital abnormality	340 (1.70)	283 (1.41)	1.34 (1.09, 1.66)	NA
	Infant mortality	974 (4.90)	898 (4.51)	1.22 (1.05, 1.41)	1.37 (1.17, 1.62)
	*Neonatal mortality*	*754* (*3.79*)	*703* (*3.53*)	*1.27* (*1.04*, *1.56*)	*1.58* (*1.25*, *2.01*)
	Low 5-minute Apgar score (<7)	1193 (6.00)	1183 (5.94)	1.05 (0.93, 1.17)	1.13 (0.99, 1.30)
	Assistant ventilation >30 minutes	818 (4.11)	799 (4.01)	1.06 (0.86, 1.30)	1.09 (0.85, 1.30)
	Respiratory distress syndrome	513 (4.35)	493 (4.18)	1.13 (0.88, 1.46)	1.09 (0.83, 1.44)
Others	Fetal mortality	35 (1.02)	34 (0.99)	1.06 (0.55, 2.05)	NA
	Congenital abnormality	67 (1.97)	44 (1.29)	2.18 (1.32, 3.61)	NA
	Infant mortality	63 (1.86)	57 (1.68)	1.24 (0.73, 2.10)	1.39 (0.77, 2.49)
	*Neonatal mortality*	*47* (*1.39*)	*44* (*1.30*)	*1.23* (*0.59*, *2.56*)	*1.60* (*0.62*, *4.15*)
	Low 5-minute Apgar score (<7)	83 (2.45)	73 (2.07)	1.49 (0.96, 2.31)	1.56 (0.97, 2.49)
	Assistant ventilation >30 minutes	110 (3.25)	101 (2.98)	1.31 (0.81, 2.13)	1.26 (0.76, 2.10)
	Respiratory distress syndrome	69 (4.10)	70 (4.16)	0.93 (0.45, 1.93)	1.01 (0.54, 2.30)

OR: Odds Ratio; aOR: Odds Ratio adjusted by birth weight, birth order and methods of delivery.

## Discussion

In this large population-based sample of mixed-gender twins, we demonstrated that male twins had significant greater odds of infant mortality and neonatal morbidity than their female co-twins. Our study included the largest population of mixed-gender twins evaluated thus far, and the results of our study contribute further evidences regarding gender-related differences in infant mortality and neonatal morbidity.

Mondal D *et al*. reviewed more than 30 million births worldwide in a meta-analysis and found that the risk of stillbirth was approximately 10% greater higher in males^13^. Our findings were in contrast with the results of this meta-analysis, as we did not identify a significant gender difference in the rate of fetal mortality. This difference may have been identified because Mondal D’s analysis included a population of singleton infants, which indicates that the presence of maternal conditions such as pregnancy obesity; low maternal socioeconomic status; and advanced maternal age might be associated with increased risk of stillbirth^14^. However, in our study population, male and female subjects had identical maternal environments. We speculated that our results may better indicate the influence of sex on the rate of fetal mortality. Moreover, our results were partially in accordance with those of the studies conducted by Steen EE *et al*. and Mullah ZD *et al*., both of which suggested the incidence of RDS, respiratory morbidity and neonatal mortality to be higher in male twins^3, 5^.

Explanations for the excess mortality and morbidity observed in males have been proposed in many singleton studies, and theorized to represent a “male disadvantage”^4, 15, 16^, suggesting that male sex seemed to be more vulnerable to mortality and morbidity, including low Apgar score, intrauterine growth restriction, respiratory insufficiency or prematurity^4, 6, 17, 18^. The underlying mechanisms contributing to the observed male disadvantage have not been elucidated. At present, some hypotheses have been proposed regarding the role of gender-associated genetic and endocrine differences in the determination of neonatal mortality or morbidity.

Authors of previous studies have speculated that the male disadvantage might be caused by hormonal environment differences. Male fetuses have been reported to have a higher level of circulating testosterone than female fetuses^19^. This difference might be associated with differences in pulmonary biomechanics and vascular development that lead to increased respiratory and neurological morbidity among preterm male neonates^20^. Currently, pulmonary disease and its complications remain predominant causes of early death^21^. The results of a study conducted by Shinwell *et al*. showed that the rates of RDS and bronchopulmonary dysplasia were significantly higher in male-male twins because of the inhibitory effects of androgens on lung development^21^. On the other hand, Mc Curnin DC *et al*.^22^ found that estrogen might be play an important role in pulmonary function and caused a persistent decrease in ventilatory support requirements in their animal study, which could explain the lower rate of assistant ventilation identified among female infants in our study. Moreover, other studies have reported that preterm females had significantly higher catecholamine levels than preterm males, which has been identified as an important defense mechanism utilized by the hypoxic fetus^23, 24^. In another animal study, Ishak N *et al*.^25^ found that male fetuses tended to have significantly lower pH levels and higher arterial partial pressure of carbon dioxide (PaCO2), lactate levels, glucose levels, and mean arterial pressures than females one hour after delivery, which may be associated with increased mortality in males. Moreover, the authors found that preterm male lambs had lower lung compliance than their female counterparts, which may have been identified due to altered surfactant phospholipid composition and function. These changes may compromise gas exchange and impair respiratory adaptation after preterm birth among males.

Authors have also hypothesized that the higher rate of mortality observed among male fetuses may be linked to differences in sex chromosomes. The Y chromosome contains only 50 genes, including the MSY gene, whereas the X chromosome contains roughly 3000 genes^26^. The Y chromosome is highly repetitive and mostly non-functional. Therefore, authors have suggested persons with XY chromosomes may be more susceptible to X-linked recessive disorders than persons with XX chromosomes; thus, male children may be less likely to be healthy than their female counterparts^27^. In our study, we found that male twins had higher rates of congenital abnormalities than their female co-twins, which might have been identified due to this chromosomal difference. Furthermore, congenital anomalies have been identified as causative factors contributing to the fetal and neonatal mortality^28^. Therefore, the risk of neonatal and infant mortality may be higher among male than female twins.

In summary, the current study demonstrated that the odds of neonatal mortality and other perinatal outcomes were higher among male twins than their female co-twins in mixed-gender pairs. Although this finding is partly in agreement with those of previous reports, a major strength of this study was the homogeneity of the study population. In this study, we focused on a cohort of mixed-gender twins, thus eliminating, to a large degree, the potentially confounding effects of iatrogenic, genetic, and/or social factors. Our data contribute to the body of evidence regarding the role of fetal sex as an important risk factor for infant mortality and neonatal morbidity in mixed-gender twins.
